# Quercetin Derivatives as Potential Therapeutic Agents: An Updated Perspective on the Treatment of Nicotine-Induced Non-Small Cell Lung Cancer

**DOI:** 10.3390/ijms242015208

**Published:** 2023-10-15

**Authors:** Naser A. Alsharairi

**Affiliations:** Heart, Mind and Body Research Group, Griffith University, Gold Coast, QLD 4222, Australia; naser.alsharairi@gmail.com

**Keywords:** flavonoids, flavonols, quercetin derivatives, non-small cell lung cancer, nicotine, molecular mechanisms

## Abstract

Flavonoids are the largest group of polyphenols, represented by many compounds that exhibit high anticancer properties. Quercetin (Q) and its main derivatives (rutin, quercitrin, isoquercitrin, isorhamnetin, tamarixetin, rhamnetin, and hyperoside) in the class of flavonols have been documented to exert anticancer activity. Q has been shown to be useful in the treatment of non-small cell lung cancer (NSCLC), as demonstrated by in vitro/in vivo studies, due to its antitumor, anti-inflammatory, anti-proliferative, anti-angiogenesis, and apoptotic properties. Some flavonoids (flavone, anthocyanins, and proanthocyanidins) have been demonstrated to be effective in nicotine-induced NSCLC treatment. However, the molecular mechanisms of quercetin derivatives (QDs) in nicotine-induced NSCLC treatment remain unclear. Thus, this review aims to summarize the available literature on the therapeutic effects of QDs in nicotine-induced NSCLC.

## 1. Introduction

Lung cancer (LC) is considered the largest contributor to cancer deaths worldwide [1]. LC consists of small cell lung cancer (SCLC) and non-small cell lung cancer (NSCLC), which are the main histological types associated with smoking. SCLC represents about 15% of all LC cases, while NSCLC accounts for a large fraction of cases (85%), and includes three subtypes: adenocarcinoma, large cell carcinoma, and squamous cell carcinoma [2,3]. 

Cigarette smoke contains a large number of carcinogenic compounds, including nitrosamines (nitrosonornicotine, NNN; and 4-methylnitrosamino-1-3-pyridyl-1-butanone, NNK), which are implicated in increasing the risk of NSCLC [4]. Nicotine is not regarded as a carcinogenic compound in tobacco and cigarette smoke, but it has the potential to facilitate tumorigenesis [5,6]. Nicotine and NNK increase the NSCLC risk by upregulating a network of signaling pathways facilitating proliferation, metastasis, and angiogenesis, and inhibiting apoptosis through activation of the nicotinic acetylcholine receptor (α7nAChRs) and beta-adrenergic receptor (β-AdrR) [6,7,8]. 

**Figure 1:**
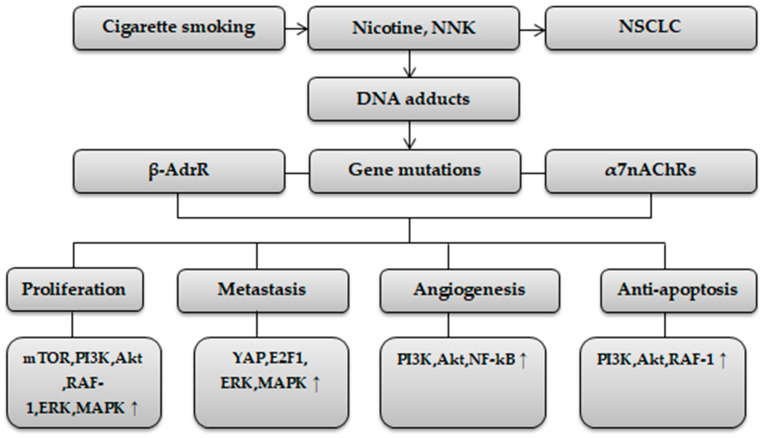
Nicotine and NSCLC development [8,9], (↑) increase.

The mechanisms by which nicotine mediates cellular processes in NSCLC have been previously described [8,9]. Figure 1 shows the mechanisms of nicotine in NSCLC development.

There is still no clear evidence confirming that dietary supplements for use in treating NSCLC are safe and effective [10,11]. The efficacy of dietary antioxidant intake in the treatment of NSCLC is also uncertain. Human and animal studies have demonstrated a beneficial effect of dietary vitamins (C, D, E, and carotenoids) and minerals (zinc and copper) intake in regards to NSCLC in smokers, while iron and retinol showed a detrimental effect [12]. Several randomized controlled trials (RCTs) of monoclonal antibody-based immunotherapy in combination with chemotherapeutic agents have shown promising therapeutic outcomes in patients with NSCLC [13]. Furthermore, there is little evidence to suggest that natural flavonoid compounds (e.g., flavone, anthocyanins, and proanthocyanidins) in medicinal plants could be effective in nicotine-induced NSCLC treatment [14,15]. Thus, there is a need for further investigations on the treatment of nicotine-induced NSCLC using other natural flavonoids.

Quercetin (Q) and its glycoside isoquercitrin (IQ) and rutin (RU) are natural flavonols found mostly in fruits, and are considered as positive allosteric modulators (PAMs)/selective agonists of human α7nAChR, which makes them potential therapeutic agents in nicotine-induced NSCLC [16,17]. Several binding sites for Q are located at the active site of α7nAChR. Q has five hydroxyl (OH) groups at the C 3, 5, 7, 3′, and 4′ positions, with two OH groups on the A and B rings and one OH group on the C ring [17]. Q acts as a potential inhibitory agent for receptor tyrosine kinase (RTK) through suppression of epidermal growth factor receptor (EGFR), which is involved in LC, by promoting interactions between the A and C rings of Q with the phosphate and binding regions in the adenosine 5′-triphosphate (ATP) binding site of EGFR, thereby producing hydrogen, π-π, and hydrophobic bonds with the phenol/aspartate of the Asp-Phe-Gly (DFG) motif in its catalytic/activation loop [18]. 

A recent review of human and animal studies showed that Q has therapeutic potential against NSCLC cells. Treatment with Q demonstrated inhibition of cell migration/invasion of A549 and HCC827 cells via suppression of Snail-independent A disintegrin and metalloprotease 9 (ADAM9) and activation of the threonine kinase (Akt) signaling pathway [19]. Q also induces apoptosis and cell cycle arrest via c-JUN NH2-terminal kinase/nuclear transcription factor-kappa B/Akt (JNK/NF-kB/Akt) signal pathway inhibition and upregulating the expression of Bcl-2-associated X protein (Bax), cleaved caspase-3, cyclin B1, p21/53 proteins, and downregulating the B-cell lymphoma-2 (Bcl-2) cascade mediated by EGFR in NSCLC cells [19,20,21,22]. Although Q is considered a potential therapeutic target against NSCLC, it has low bioavailability in humans due to its instability and poor water solubility and permeability [23]. Many drug delivery approaches have been recommended to enhance Q bioavailability, including nanoparticles, micelles, inclusion complexes, and liposomes [24]. Liposomal Q has been found to induce cell apoptosis and suppress tumor growth both in vitro and in vivo [25]. A study has shown that Q increases green tea polyphenols (GTPs) bioavailability and decreases their methylation in A549 cells. That study reported a high anti-proliferative effect of Q in combination with GTPs and epigallocatechin gallate through multidrug resistance proteins (MRPs) and catechol-O-methyltransferase (COMT) inactivation [26].

**Figure 2:**
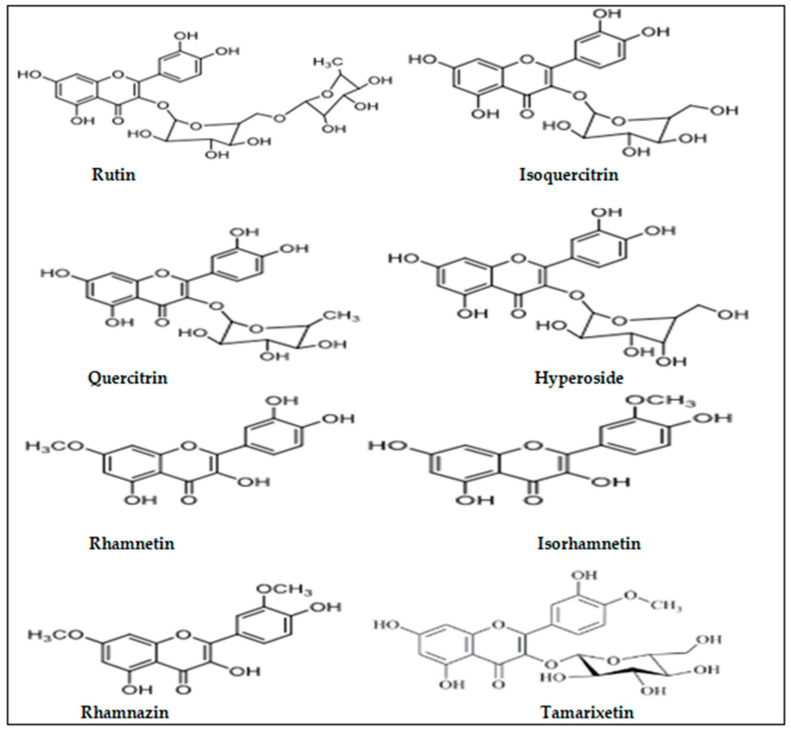
Chemical structures of QDs [27,28,29,30].

Given that Q has proven to be effective for NSCLC treatment, targeting its derivatives might help in understanding the α7nAChRs-mediated signaling pathways as potential targets for NSCLC treatment. Because there have been no reviews on the mechanisms of quercetin derivatives (QDs) for nicotine-induced NSCLC treatment to date, this review aims to highlight the therapeutic effects of QDs (rutin, quercitrin, isoquercitrin, isorhamnetin, tamarixetin, rhamnetin, rhamanzin, and hyperoside) via various mechanisms of action. Each derivative has a unique chemical structure [27,28,29,30] that may exhibit a high inhibitory effect against nicotine-induced NSCLC. The chemical structures of these QDs are presented in Figure 2.

## 2. Methods

A literature search of studies published in the English language was conducted from their inception up to September 2023 through the PubMed/MEDLINE database. The search used the following keywords: “QDs/metabolites” OR “rutin” OR “quercitrin” OR “isoquercitrin” OR “isorhamnetin” OR “tamarixetin” OR “rhamnetin” OR “hyperoside” AND “lung cancer” OR “NSCLC” AND “molecular mechanism” AND “nicotine.” All studies with a primary focus on QDs were included, and the search was not limited to a particular study design. Studies focusing on Q as a potential target for the treatment of NSCLC were not considered. The search identified 155 studies for possible inclusion. As a result, 25 studies met the search criteria.

## 3. Quercetin Derivatives in Nicotine-Induced NSCLC Therapy

### 3.1. Rutin

RU (3,3′,4′,5,7-pentahydroxyflavone-3-rhamnoglucoside), also known as Q-3-*O*-rutinoside, is a flavonol compound found in various fruits and vegetables. It is less bioavailable and soluble than Q in humans [31]. RU is also absorbed more slowly than Q in the small intestine of rats [32]. 

RU could be useful in the treatment of NSCLC mediated by α7nAChR. RU and Q act on α7nAChR-dependent ion currents. Q induces the enhancement of ACh-induced inward peak currents (*I_ACh_*) on cells expressing human α7nAChR and increases the extracellular Ca^2+^ level-mediated potentiation of *I_Ach_* via interactions with Ca^2+^-binding sites for α7nAChR in *Xenopus oocytes* (a system for the expression of plasma membrane transport proteins) [33]. Q exhibits differential regulation of α7nAChR channel activity with respect to RU in such a way that Q increases *I_ACh_*, while RU decreases *I_ACh_* in *Xenopus oocytes* expressing human α7AChR, and such effects exist in a voltage-insensitive and non-competitive manner. Q-mediated *I_ACh_* suppression can be improved when Q is co-administered with RU, suggesting that RU may have a significant role in the regulation of α7nAChR [34]. 

RU was found to inhibit β-amyloid (Aβ) peptide-induced neuronal cytotoxicity, nitric oxide (NO), and the production of reactive oxygen species (ROS) and proinflammatory cytokines by reducing interleukin (IL)-1β and tumor necrosis factor (TNFα) production in microglia [35]. Plasma levels of Aβ were found to be significantly increased in patients with multiple cancers, including LC [36].

An in vitro study has shown that α-L-rhamnosidase (α-R) cleaves terminal α-rhamnose in flavonoid rutinosides/glycosides (hesperidin, naringin, diosmin, and troxerutin), which increases the anti-proliferative and anti-oxidant activities of RU against various cancer cell lines, including NSCLC (H460) cells [37]. In contrast, β-glucosidase (β-G) has been shown to exert high potency against LC tumor growth. β-G converts rutin to rutinose disaccharide and Q through its ability to remove sugar moieties such as glucose linked to flavonoids in positions 3 and 7 of the C ring [38]. β-G has been described as the key glycoprotein-processing enzyme that inhibits the expression of p53 in NSCLC cells [39]. Knockout of β-G was found to inhibit cell migration/metastasis and induce apoptosis and/or autophagy in NSCLC cells through the suppression of RTK signaling pathways [40]. 

α-R may convert RU into IQ with high bioactivity and bioavailability by combining with β-G [41]. Thus, α-R coupled with β-G provides high bioactive effects, which inhibit the proliferation and induction of apoptosis in NSCLC cells. Recent molecular docking studies showed that the OH groups of RU (C7-OH) form stable H-bond interactions with several amino acid residues, including serine, aspartic acid, phenylalanine, glutamine, glutamic acid, and arginine [42]. Therefore, these OH groups may create interactions with amino acid residues in the binding pocket of α7nAChR, displaying complex modulation of the receptor. 

A few studies investigating the therapeutic efficacy of RU in nicotine-induced NSCLC showed that the mechanisms underlying its effects have not been explored. Transformation of rutin to quercetin-3-β-D-glucoside (Q3G) using α-R and β-G from a crude enzyme extract of *Aspergillus niger* (*A. niger*) has been shown to inhibit NSCLC cell proliferation [43]. A study has demonstrated that RU-loaded liquid crystalline nanoparticles exert anti-proliferative/anti-migratory and apoptotic effects on NSCLC cells [44]. RU reduces superoxide anion/intracellular ROS production and suppresses the proliferation and migration/adhesion of NSCLC cells, although it showed cytotoxic effects at concentrations higher than 500 µM (IC_50_ = 559.83 µM) [45]. RU from *Artemesia judaica* L. (*A. judaica* L.) ethanolic extract showed apoptotic potential against NSCLC cells and arrested the cell cycle at the G2/M phase. A cytotoxic activity of *A. judaica* L. extract was reported against A549 cells (IC_50_ = 14.2 μg/mL). This may be due to the presence of several extracts of different polarities [46]. RU has shown antioxidant effects on NSCLC cells by decreasing NNK-induced intracellular ROS and inhibiting the DNA damage induced by β-carotene [47]. Thus, RU may have therapeutic potential in nicotine-induced NSCLC cells by inhibiting proliferation, migration, and adhesion, and promoting apoptosis. RU also has a beneficial effect as a potential antioxidant in inhibiting NNK-induced DNA damage in NSCLC cells.

### 3.2. Isorhamnetin

Isorhamnetin (IS) (3′-methoxy-3,4′,5,7-tetrahydroxyflavone), a flavonol compound found in the leaves of medicinal plants such as *Hippophae rhamnoides* L. (*H. rhamnoides* L.) and *Ginkgo biloba* L. (*G. biloba* L.) has a wide range of therapeutic effects against several diseases, such as cerebrovascular diseases and atherosclerosis, It has also shown anti-tumor effects against various common cancers, including LC [48]. A few in vivo studies so far have shown a high IS bioavailability. Phytic acid improves the oral absorption of flavone compounds of *H. rhamnoides* L, including IS [49]. The bioavailability of IS increased when it was coingested with *G. biloba* L. extract solid dispersions and phospholipid complexes [50]. 

The therapeutic effects of IS against nicotine-induced NSCLC may be related to the inhibition of α7nAChR and its downstream signaling pathways. However, whether IS has potential α7-PAM activity remains unknown. IS is structurally similar to Q, which has an OH group at C-3. IS has four OH groups at the C-3,5,7,4′ positions, with one OH group on the B and C rings and two OH groups on the A ring [27,28]. IS also has one methoxy group in the C-5′ position on the B ring [27,28]. Thus, IS may be an effective anti-NSCLC agent by binding to the molecules involved in the α7nAChR-mediated signaling pathways. 

Another possible explanation for the anti-tumor activities of IS against nicotine-induced NSCLC might be due to the enzymatic de-glycosylation of IS by α-R and β-G [51], which could lead to improved efficacy of IS for the treatment of NSCLC. IS may interact with the active site of both α-R and β-G, resulting in the inhibition of signaling pathway regulation of cellular processes implicated in nicotine-induced NSCLC.

IS may have anti-proliferative and apoptotic/autophagic effects against nicotine-induced NSCLC via regulation of α7nAChR and its downstream signaling pathways. IS inhibits proliferation/colony formation ability and promotes the apoptosis/autophagy of NSCLC cells in a time and dose-dependent manner via the mitochondria-dependent apoptosis pathway [52]. Treatment with IS demonstrated significant inhibition of migration and invasion via inactivation of the oncogenic kinase signaling pathway [53]. 

### 3.3. Hyperoside

Hyperoside (HP), also termed Q 3-*O*-β-D-galactopyranoside, a naturally occurring flavonol that is widely present in plants such as *Polygonum aviculare*, *Crataegus pinnatifida,* and *Hypericum monogynum*, exerts a wide range of anticancer effects [29,54]. The therapeutic effect of HP on nicotine-induced NSCLC is still unclear, but it may be attributed to its aglycone Q, which has a great binding affinity for human α7nAChR [29]. HP may have high affinity and potential for binding to the active site of α7nAChR. The anti-NSCLC activity of HP may depend on its having eight OH groups on the A, B, and C-rings of their structure, with two OH groups on the B ring (positions 3′ and 4′), two OH groups on the A ring (positions 5 and 7), and four OH groups in the glycosides linked to the C ring (positions 2″, 3″, 4″, and 5″) [27,29]. In vivo, low HP bioavailability may be due to its poor oral absorption [55,56].

A few studies suggest that HP treatment exhibits a range of effects against nicotine-induced NSCLC, including anti-proliferative, anti-migration, anti-invasion, anti-inflammatory, and apoptotic/autophagic activities. HP has been reported to suppress proliferation and promote apoptosis of T790M-positive NSCLC cells by increasing forkhead box protein O1 (FoxO1) expression in colon cancer-associated transcript 1 (CCAT1)-knockdown NSCLC cells [57]. HP inhibits the expression of genes associated with tumor migration and invasion in NSCLC cells by suppressing signaling pathways involving tumor metastatic genes [58]. HP decreases viability and induces apoptosis of NSCLC cells by upregulating pro-apoptotic-related gene expression through activation of apoptotic pathways [59]. HP activates apoptosis and autophagy in proliferating NSCLC cells by increasing apoptotic/autophagic-related gene expression via a range of signaling pathways associated with tumorigenesis [60]. HP regulates genes involved in apoptosis while downregulating genes involved in proliferation, migration, invasion, and inflammation of NSCLC cells via inhibiting the NF-kB signaling pathway [61]. HP inhibits proliferation, increases apoptosis, and causes arrested growth of NSCLC cells at the G1/S phase by decreasing the protein expression of cyclin-dependent kinase (CCND1) and coding sequence (CDK 4 & 6) through its interaction with the microRNA-let7a-5p [62]. HP significantly inhibited proliferation, migration, invasion, and angiogenesis; induced apoptosis; and arrested the cell cycle at the S phase in NSCLC cells, mediated through upregulating pro-apoptotic and downregulating anti-apoptotic protein levels [63]. HP reduces the inflammation of NSCLC cells by decreasing *Mycoplasma pneumoniae pneumonia* (MPP)-induced pro-inflammatory cytokines production through NF-κB signaling pathway inactivation [64]. HP reduces hypoxia-induced viability and proliferation of NSCLC cells, as demonstrated by upregulating the expression of heme oxygenase-1 (HO-1) through activating AMP-activated protein kinase (AMPK) [65].

### 3.4. Rhamnetin and Rhamnazin

Rhamnetin (RT) and rhamnazin (RZ) are methylated QDs distributed widely in fruits and vegetables that exert antibacterial, anti-inflammatory, antioxidant, and anticancer properties [27,66]. Previous evidence has demonstrated that the methoxy groups produce steric hindrance and reduce the free radical scavenging ability of flavonoids. However, the OH groups endow flavonols with high radical scavenging capability [67]. RT and RZ are structurally similar to IS but differ from other flavonols by having methoxy groups on the A and B rings [27,28]. RT (7′-*O*-methoxy Q) contains a methyl group at the 7′ position on the A ring, while RZ (3′,7′-dimethylquercetin) has two methyl groups at the 3 and 7′ positions on the A and B rings [27,30]. RT and RZ have an OH group at C-3, which plays a significant role in anti-tumor activity against nicotine-induced NSCLC. RT possesses four OH groups at C-5, 3, 3′, and 4′, with one OH group on the A and C rings and two OH groups on the B ring. RZ has three OH groups in its structure at the 5 position on the A ring, the 3 position on the C ring, and the 4′ position on the B ring [27,30]. 

RT has been shown to displace a selective α7nAChR ligand, methyllycaconitine [^3^H]-MLA, or an αβ2 selective ligand, [^3^H]-cytisine, with the lowest cytotoxicity activity (IC_50_) of nicotine, resulting in inhibited NO and TNFα release [68]. RT also exerts α7nAChRs agonist activity in vivo, as demonstrated by reduced excitotoxicity following ethanol exposure and neuroinflammation by decreasing lipopolysaccharide (LPS)-induced TNFα and NO production [69].

RZ may have a significant role in anti-angiogenic effects in NSCLC cells, as demonstrated by a xenograft mouse model. RZ suppresses in vivo angiogenesis mediated by NSCLC cells by inhibiting vascular endothelial growth factor A (VEGFA) and vascular endothelial growth factor receptor 2 (VEGFR2) phosphorylation through anti-programmed cell death 1/programmed cell death ligand 1(PD-1/PD-L1) signaling pathway inactivation. Treatment with RZ (20 μM) and anti-CD3 antibody (1 μg/mL) exhibits cytotoxic effects on NSCLC cells, promotes T cell proliferation, and increases the production of cytotoxic mediators [70]. 

### 3.5. Quercitrin

Quercitrin (QU), also known as Q-3-*O*-rhamnoside, is a Q derivative that is structurally similar to HP. QU has seven OH groups on the A, B, and C rings, with two OH groups on the A ring (positions 5 and 7), two OH groups on the B ring (positions 3′ and 4′) and three OH groups in the glycosides linked to the C ring. QU differs from QDs by having one methyl group in the glycosides linked to the C ring, which makes its anti-NSCLC ability lower than the other derivatives [27,30]. Thus, Qu would be expected to have lower levels of effectiveness as an anti-NSCLC agent than other QDs in binding to the α7nAChR-mediated signaling pathways.

A few studies have demonstrated that QU exerts anti-proliferative, anti-migration/invasion, and apoptotic effects on NSCLC cells. QU inhibits proliferation and induces apoptosis in NSCLC cells via increasing caspase-3 enzyme activity and the nucleosomal enrichment factor, and inducing the loss of mitochondrial membrane potential. QU exerts cytotoxic effects on NSCLC cells in a dose-dependent manner (50 μmol), which may be due to inducing lactate dehydrogenase (LDH) release to baseline levels of toxicity [71]. NSCLC cell migration and invasion has been shown to be reduced by QU treatment at different concentrations by inhibiting the transcript levels of Gap Junction Protein Beta 2 (GJB2) in these cells [72]. 

### 3.6. Tamarixetin

Tamarixetin (TA), also called 4′-*O*-methoxy Q, is a derivative that has four OH groups on C 3, 5, 7, and 3′ [30], among which the OH group at C-3 may improve the anti-tumor activity against NSCLC [67]. However, TA has a methyl group at the 4′ position on the B ring [30]. Only one study showed that TA and IS inhibit the proliferation of NSCLC cells. This inhibition was accompanied by increased apoptosis-related gene expression. Cytotoxicity analysis has indicated cytotoxic activity of TA and IS with an IC_50_ value between 15–26 μM in NSCLC cells. This may be due to the combination of different flavonoids, which increases the cytotoxic potencies of these methylquercetins [73]. 

Table 1 summarizes the in vitro and in vivo studies that have evaluated QDs in nicotine-induced NSCLC treatment. 

## 4. Quercetin Derivatives in Combination with Chemotherapeutics/Radiotherapy in Nicotine-Induced NSCLC Therapy

A few studies have proved that RU and IS inhibit proliferation and enhance cisplatin/carboplatin-induced apoptosis in NSCLC cells. Treatment with RU extracted from *Urtica dioica* L. in combination with cisplatin suppresses proliferation of NSCLC by promoting endoplasmic reticulum (ER) stress-induced apoptosis through DNA damage-inducible gene 153 (GADD153) activation. RU is safe on NSCLC cells, capable of antagonizing cisplatin cytotoxicity at different doses (25, 50, 75, and 100 μg/mL) [74]. RU in combination with cisplatin showed potent apoptotic effects in NSCLC cells by increasing the expression of TNF-α and glycogen synthase kinase (GSK)-3β [75]. A study reported carboplatin and a cisplatin/IS combination as being effective in inhibiting proliferation and inducing apoptosis in NSCLC cells via mitochondrial apoptotic pathway activation [76]. IS in combination with cisplatin results in a significant inhibition of proliferation and induction of apoptosis/cell cycle arrest in NSCLC cells. This was demonstrated via activation of apoptotic genes and suppression of oncogenes genes [77]. 

Studies demonstrated that IS and RT inhibit proliferation and induce NSCLC cell apoptosis during irradiation (IR). NSCLC cells treated with IS and IR results in the inhibition of proliferation and the induction of apoptosis by increasing/decreasing apoptosis-related protein expression via inactivation of IR-induced-NF-κB and activation of IR-induced-interleukin (IL)-13 signaling pathways. The results also demonstrated the IS/IR combination had no cytotoxic effects on NSCLC cells [78]. RT and cirsiliol-IR combination treatment exhibits anti-proliferative and apoptotic effects on NSCLC cells by reducing epithelial–mesenchymal transition (EMT)-related molecules and increasing tumor-suppressive miRNAs [79]. 

Table 2 summarizes the in vitro and in vivo studies that have assessed QDs in combination with cisplatin/carboplatin and IR in nicotine-induced NSCLC treatment.

## 5. Limitations

Most of these studies were conducted on in vitro models, and studies on humans are lacking. Using different dosage regimens of QDs in vitro and in vivo may lead to problems in design-ideal treatment strategies for nicotine-induced NSCLC. Treatment with different concentrations may affect the cellular and molecular mechanisms underlying the therapeutic potential of QDs in nicotine-induced NSCLC. The potential mechanisms to explain RU therapeutic effects against nicotine-induced NSCLC are unclear and have not been investigated yet. Although RU, RZ, QU, and TA are considered cytotoxic to NSCLC cells at specific concentrations, they do not harm these cells and could be ideal compounds for nicotine-induced NSCLC treatment through several mechanisms or used as adjuvant agents in combination with chemotherapeutic drugs or IR.

## 6. Conclusions

Q has major derivatives that exist in fruits and vegetables and are in the class of flavonols with anticancer properties. However, the mechanisms by which QDs exert therapeutic effects against nicotine-induced NSCLC are not fully understood. QDs may have the ability to alter the activity of α7nAChRs-mediated signaling pathways involved in cellular processes driving the development of NSCLC. Treatment with all QDs exhibits a range of therapeutic effects in nicotine-induced NSCLC, including anti-proliferative, anti-migration, anti-invasive, anti-viability, apoptotic, and autophagic activities. HP showed anti-inflammatory and anti-angiogenesis effects, while RZ demonstrated anti-angiogenesis activity in nicotine-induced NSCLC.

RU, IS, and RT exert anti-proliferative and apoptotic effects in combination with chemotherapeutic agents and/or IR as a treatment for nicotine-induced NSCLC cells. RU and IS reduce cisplatin/carboplatin cytotoxicity by inhibiting proliferation and inducing apoptosis of NSCLC cells through different cellular signaling pathways. IS and RT increase apoptosis in nicotine-induced NSCLC cells, which was further enhanced by IR treatment. 

## 7. Future Directions

QDs serve as promising treatment agents in targeting nicotine-induced NSCLC. Thus, clinical applications of these derivatives in NSCLC trials are needed. The studies presented suggest the toxic effect of some QDs is accompanied by no harm to nicotine-induced NSCLC cells. Future studies in humans are warranted to examine whether the toxicity of QDs would decrease the effectiveness of these therapeutic agents for nicotine-induced NSCLC. Further studies are needed to investigate the effects of QDs in combination with chemotherapeutic agents and IR in nicotine-induced NSCLC treatment. Further studies are also needed to explore the accurate dosage of QDs in treatment. Future directions for exploring potential mechanisms underlying the therapeutic effects of QDs in nicotine-induced NSCLC, particularly in smokers, are warranted.

## Figures and Tables

**Table 1:** The role of QDs in nicotine-induced NSCLC therapy.

Study Design	QDs	Cell Line	Dosage	Activity	Target Molecular Genes	Target Mechanisms	Ref.
In vitro	RU	A549	0, 50, 100, 150, 200, 250 μM	Anti-proliferative	NA	NA	[43]
In vitro	RU	A549	2.5, 5, 10, 20 μM	Anti-proliferative, anti-migration, apoptosis	MMP-9 ↓	NA	[44]
In vitro	RU	A549	31.25, 62.5, 125, 250, 500, 1000 μM	Anti-proliferative, anti-migration, anti-adhesion	ROS ↓	NA	[45]
In vitro/vivo	RU	A549	0.1, 1, 10, 100 μg/mL (in vitro) 100 mg/kg BW/day of *A. judaica* L. (in vivo)	Apoptosis, G2/M phase cell cycle arrest	Caspase-3/8/9, P53 Bax ↑ Bcl-2 ↓	NA	[46]
In vitro/vivo	IS	A549	0–16 μM (in vitro) 0.5 mg/kg BW/day (in vivo)	Anti-proliferative, apoptosis, autophagy	Caspase-3/9, cytochrome *C*, Bax, P53, c-PARP, Beclin1, LC3-II ↑	Mitochondria-dependent caspase ↑	[52]
In vitro	IS	A549	2.5, 5, 10 μM	Anti-migration, anti-adhesion, anti-invasion	E-cadherin ↑ MMP2/9, snail, vimentin, N-cadherin ↓	Akt/ERK1/2 ↓	[53]
In vitro/vivo	HP	H1975	0–150 μM (in vitro) 25 mg/kg BW/day (in vivo)	Anti-proliferative, apoptosis	FoxO1 ↑	CCAT1 ↓	[57]
In vitro	HP	A549	1, 2, 5 μM	Anti-migration, anti-invasion	MMP-2/9 TIMP-2 ↓	PI3K/Akt, p38 MAPK ↓	[58]
In vitro	HP	A549	10, 50, 100 μM	Apoptosis, anti-viability	Caspase- 3/9, cytochrome *c*, apoptosis-inducing factor ↑	p38 MAPK, JNK ↑	[59]
In vitro	HP	A549	0.5, 1, 2 mmol/L	Apoptosis, autophagy	Caspase- 3, c-PARP, LC3-II ↑	Akt, mTOR, p70S6K. 4E-BP1 ↓ ERK1/2 ↑	[60]
In vivo	HP	A549	15, 20, 25 mg/kg BW/for a month	Anti-proliferative, anti-migration, anti-invasion, anti-inflammatory, apoptosis	Caspase- 3, Bax ↑ MMP-2/9, Bcl-2, IL-6, IL-18, IL-1β, TNF-α ↓	NF-kB ↓	[61]
In vitro	HP	A549	0, 10, 20, 50, 100, 200, 400 μg/mL	Anti-proliferative, apoptosis, G1/S phase cell cycle arrest	CCND1, CDK-4/6 ↓	NA	[62]
In vitro/vivo	HP	A549, H1975	0, 20, 40, 60, 80, 100 μg/mL (in vitro) 20, 40, 80 μg/mL (in vivo)	Anti-proliferative, anti-migration, anti- invasion, anti-angiogenic, apoptosis, S phase cell cycle arrest	Caspase- 3/9, c-PARP, Bax, Bad, Bak, Cyto-c, Apaf-1, p53 ↑ MMP-2/7, c-Myc, Cyclin-D1, CDK1, Akt, Bcl-2, Bcl-xl ↓	NF-kB ↓	[63]
In vitro	HP	A549	0, 25, 50, 100, 200, 400, 1000 μg/ml	Anti-inflammatory	IL-8, TNF-α, CCR4, CCL5, p-NF-κB-p65 ↓	NF-kB ↓	[64]
In vitro	HP	A549	10, 50, 100 μg/ml	Anti-proliferative, anti-viability	HO-1, ROS ↑ GSH ↓	AMPK ↑	[65]
In vivo	RZ	H1975	200 mg/kg BW/day	anti-angiogenic	VEGFA, VEGFR2 ↓	PD-1/PD-L1 ↓	[70]
In vitro	QU	A549, H358	5, 10, 25, 50 μM	Anti-proliferative, apoptosis	Caspase-3, loss of mitochondrial membrane potential ↑	Phosphatidylinositol signaling system, leukocyte transendothelial migration, cell adhesion ↓	[71]
In vitro	QU	H1299, H1650	2, 5, 10 μM	Anti-migration, anti-invasion	GJB2 ↓	NA	[72]
In vitro	TA, IS	A549, HCC-44	15–26 μM	Anti-proliferative, apoptosis	Caspase- 3/9 ↑	Intrinsic and extrinsic apoptotic pathway- caspase cascades ↑	[73]

(↓) decrease, (↑) increase; NA = not available.

**Table 2:** The role of QDs in combination with radiotherapy/chemotherapy in nicotine-induced NSCLC therapy.

Study Design	QDs	Cell Line	Dosage	Activity	Target Molecular Genes	Target Mechanisms	Ref.
In vitro	RU	A549, H1299, H460, H322	10–100 μg/mL (*Urtica dioica* L.)	Anti-proliferative, apoptosis	Caspase- 3/8, c-PARP ↑	GADD153 ↑	[74]
In vitro	RU	A549	1 × 10^−8^ (low group), 2 × 10^−8^ (medium group) or 4 × 10^−8^ mol/L (high group)	Apoptosis	TNF-α, GSK-3β ↑	NA	[75]
In vitro	IS	A549	0, 2.5, 5, 25, 50, 100 μM	Anti-proliferative, apoptosis, G2/M phase cell cycle arrest	Caspase-3/9, PARP ↑	Mitochondria-mediated caspase ↑	[76]
In vitro/vivo	IS	A549	20, 40, 80 μg/mL (in vitro) 50 mg/kg/day (in vivo)	Anti-proliferative, apoptosis, S and G0/G1 phase cell cycle arrest	Caspase-3, Bax, P53 ↑ Bcl-2, cyclinD1, PCNA ↓	NA	[77]
In vitro	IS	A549, H460	5, 10, 20, 40, 60, 80 μM	Anti-proliferative, apoptosis	Bax ↑ Bcl2, p-IκBα, p-NF-κBp65 ↓	NF-κB ↓ IL-13 ↑	[78]
In vivo	RT	H1299, H460	200 μg/mL /kg body weight	Anti-proliferative, apoptosis	miR-34a, p53, E-cadherin ↑ NF-kB, p65, Hes-1, Hey-1, vimentin, fibronectin ↓	NF-κB, Notch-1 ↓	[79]

(↓) decrease, (↑) increase; NA = not available.

## Data Availability

Not applicable.

## Abbreviations

Aβ	β-amyloid
ADAM9	A disintegrin and a metalloprotease 9
Akt	Threonine kinase
AMPK	AMP-activated protein kinase
ATP	Adenosine 5′-Triphosphate
β-AdrR	Beta-adrenergic receptor
Bax	Bcl-2-associated X protein
Bcl-2	B-cell lymphoma-2
β-G	β-glucosidase
CCAT1	Colon cancer-associated transcript 1
CCL5	Chemokine C-C motif ligand 5
CCND1	Cyclin-dependent kinase
CCR4	Chemokine receptor 4
CDK	Coding sequence
COMT	Catechol-O-methyltransferase
E2F	E2F transcription factors 1
4E-BP1	4E-binding protein 1
EGFR	Epidermal growth factor receptor
EMT	Epithelial-mesenchymal transition
ER	Endoplasmic reticulum
ERK	Extracellular signal-regulated kinase
FoxO1	Forkhead box protein O1
GADD153	DNA damage-inducible gene 153
GJB2	Gap Junction Protein Beta 2
GSH	Glutathione
GSK	Glycogen synthase kinase
GTPs	Green tea polyphenols
HO-1	Heme oxygenase-1
HP	Hperoside
IL	Interleukin
IQ	Isoquercitrin
IR	Irradiation
IS	Isorhamnetin
JNK	c-JUN NH2-terminal kinase
LC	Lung cancer
LC3-II	LC3-phospholipid conjugate
LDH	Lactate dehydrogenase
LPS	Lipopolysaccharide
MAPK	Mitogen-activated protein kinase
MLA	Methyllycaconitine
MMP	Metalloproteinase
MRPs	Multidrug resistance proteins
mTOR	Mammalian target of rapamycin
nAChR	Nicotinic acetylcholine receptor
NF-kB	Nuclear transcription factor-kappaB
NNK	4-methylnitrosamino-1-3-pyridyl-1-butanone
NNN	Nitrosonornicotine
NO	Nitric oxide
NSCLC	Non-small cell lung cancer
OH	Hydroxyl
P38	Protein 38
PAM	Positive allosteric modulator
PARP	Poly ADP ribose polymerase
PCNA	Proliferating cell nuclear antigen
PD-1	Anti-programmed cell death 1
PD-L1	Programmed cell death ligand 1
PI3K	Phosphatidylinositol-3 kinase
Q	Quercetin
QDs	Quercetin derivatives
Q3G	Quercetin-3-β-D-glucoside
QU	Quercitrin
α-R	α-L-rhamnosidases
RAF-1	Retinoblastoma tumor suppressor protein-proto-oncogene
RCTs	Randomized controlled trials
ROS	Reactive oxygen species
RT	Rhamnetin
RTK	Receptor tyrosine kinase
RU	Rutin
RZ	Rhamnazin
SCLC	Small cell lung cancer
TA	Tamarixetin
TNF	Tumor necrosis factor
VEGFA	Vascular endothelial growth factor A
VEGFR2	Vascular endothelial growth factor receptor 2
YAP	Yes-associated protein
